# Firearm Homicide in Pregnant Women and State-Level Firearm Ownership

**DOI:** 10.1001/jamanetworkopen.2025.42447

**Published:** 2025-11-10

**Authors:** Ayesha Dholakia, Michael C. Monuteaux, Gabrielle D’Ambrosi, Suzanne G. McLone, Eric Fleegler, Lois K. Lee

**Affiliations:** 1 Division of Emergency Medicine, Boston Children’s Hospital, Boston, Massachusetts; 2 Biostatistics and Research Design Center, Boston Children’s Hospital, Boston, Massachusetts; 3 Department of Epidemiology, Boston University School of Public Health, Boston, Massachusetts; 4 MGH Gun Violence Prevention Center, Massachusetts General Hospital, Boston; 5 Department of Pediatrics, Harvard Medical School, Boston, Massachusetts; 6 Department of Emergency Medicine, Harvard Medical School, Boston, Massachusetts

## Abstract

**Importance:**

Homicide is the leading cause of death among pregnant women, mostly from firearms. State-level variability in pregnancy-associated homicide rates and their association with firearm ownership is not well characterized.

**Objectives:**

To describe pregnancy-associated firearm homicide rates across states and examine their association with state-level firearm ownership prevalence.

**Design, Setting, and Participants:**

This retrospective cross-sectional study using the National Violent Death Reporting System included women of childbearing age (15-49 years) who died from homicide in 37 states (January 1, 2018, to December 31, 2021). Women were categorized as pregnant (including in the last 12 months) vs nonpregnant. Data were analyzed between January 2023 and July 2025.

**Exposure:**

The primary exposure was state-level firearm ownership prevalence.

**Main Outcomes and Measures:**

The primary outcome was state-level pregnancy-associated firearm homicide rates. Firearm ownership prevalence was estimated using a proxy: the ratio of state firearm suicides to total suicides. Multivariable generalized estimating equations with a negative binomial link were used to estimate adjusted incident rate ratios (IRRs) with 95% CIs for the outcome of pregnancy-associated homicide, adjusting for estimated state-level firearm prevalence, year, and state-level covariates (unemployment, poverty, insurance, age, sex, and population density).

**Results:**

Among homicides in 7063 women (median [IQR] age, 30.0 [24.0-38.0] years; 153 [2.2%] American Indian or Alaska Native, 115 [1.6%] Asian or Pacific Islander, 3278 [46.4%] Black or African American, 2565 [36.3%] White, 123 [1.7%] other [other or unspecified race and >1 race], and 29 [0.4%] unknown), 434 (6.1%) occurred in pregnant women. Most homicides (250 [57.6%]) occurred among women of non-Hispanic Black race. Firearms were used in 341 homicides (78.6%) in pregnant women. Firearm homicide rates were higher in pregnant vs nonpregnant women (38.63 vs 28.20 per 1 000 000 live births; IRR, 1.37; 95% CI, 1.22-1.53). Across states, pregnancy-associated firearm homicide rates ranged from 0 to 111.43 per 1 000 000 live births. In adjusted models, the adjusted IRRs were 1.06 (95% CI, 1.03-1.09) for all-cause homicide and 1.08 (95% CI, 1.04-1.12) for firearm homicide for each 1% increase in estimated firearm ownership.

**Conclusions and Relevance:**

In this cross-sectional study of homicides among women, state-level firearm ownership was significantly associated with all-cause and firearm-specific homicide among pregnant women, independent of state-level factors. These results suggest that homicide prevention interventions should focus on women, especially during the increased risk period of pregnancy.

## Key Points

**Questions:**

How do pregnancy-associated firearm homicide rates vary across states and are they associated with state-level firearm ownership?

**Findings:**

In this cross-sectional study performed from 2018 to 2021, pregnant women experienced a 37% higher firearm homicide rate than nonpregnant women. In adjusted models, every 1% increase in state-level firearm ownership was significantly associated with a 6% increase in all-cause and 8% increase in firearm-specific homicide rates in pregnant women.

**Meaning:**

This study’s results suggest an urgent need for focused state-level interventions to protect maternal safety.

## Teaser

This cross-sectional study examines firearm homicide rates in pregnant compared with nonpregnant women and whether estimated state-level firearm ownership is associated with homicide deaths among pregnant women.

## Introduction

The US has one of the highest maternal mortality rates among high-income countries,^1^ with substantial disparities by race and ethnicity.^2^ Homicide is the leading cause of these deaths for pregnant and postpartum women,^3,4^ yet violence remains an underrecognized threat to maternal health.^5^ Both pregnant and postpartum women face an increased risk of homicide (ie, pregnancy-associated homicide)^6^ compared with their nonpregnant peers,^3,7,8,9^ with increasing homicide rates nationally.^10^ More than two-thirds of pregnancy-associated homicides are by firearms,^10,11^ and recent literature^12^ has highlighted wide variation in firearm-involved pregnancy-associated deaths across states. These disparities suggest broader structural differences, such as firearm access, may shape maternal health outcomes in different states.

In the general population, state-level firearm ownership is a substantial risk factor for firearm violence, with states with higher firearm ownership prevalence having higher rates of firearm deaths.^13,14^ Less is understood about the association between state-level firearm ownership and pregnancy-associated firearm homicide rates specifically. Given that homicide is a leading, but preventable, cause of maternal mortality, understanding how structural factors such as firearm prevalence contribute to geographic variability in pregnancy-associated homicide is critical to guide prevention efforts. This study aims to (1) describe the demographic, incident, and suspect characteristics of homicides in pregnant women; (2) examine state-level pregnancy-associated firearm homicide rates using incident-level data; and (3) analyze the association between estimated state-level firearm ownership prevalence and pregnancy-associated homicide rates with multivariable regression modeling. We hypothesized that firearm homicide rates would be higher in pregnant compared with nonpregnant women and that higher estimated state-level firearm ownership would be associated with increased pregnancy-associated homicide rates and by firearm specifically.

## Methods

### Study Design and Data Sources

We conducted a retrospective cross-sectional study using data from the National Violent Death Reporting System (NVDRS) from the Centers for Disease Control and Prevention (CDC). We included data from January 1, 2018, the first year of NVDRS data collection from all 50 states, to December 31, 2021, the most recent year of NVDRS Restricted Access Database data available at the time of analysis. This study follows the Strengthening the Reporting of Observational Studies in Epidemiology (STROBE) guidelines for cross-sectional studies. The Boston Children’s Hospital institutional review board deemed this study exempt from review, and informed consent was not required because it was considered not human subjects research.

Demographic, incident, and suspect data for all homicides in women were obtained from the NVDRS Restricted Access Database, a publicly available, multistate, case-level dataset accessed via approved application. Individuals were identified as dying by homicide as categorized by the NVDRS, which uses a multisource method to determine manner and intent of death, including obtaining information from death certificates, coroner and medical examiner reports, and law enforcement records. Data on state-level firearm suicide and total suicides, used to estimate firearm ownership,^15,16^ were obtained from the CDC’s Web-based Injury Statistics and Query Reporting System (WISQARS).^17^ Population estimates for homicides among nonpregnant women aged 15 to 49 years were also obtained from WISQARS. Live birth counts were obtained from the CDC Wide-ranging Online Data for Epidemiologic Research (WONDER) natality data files.^18^ These data were used to determine the population denominator to calculate pregnancy-associated homicide rates. Additional state-level population covariates, including percentage aged 15 to 29 years, percentage male, percentage living below the poverty level, percentage unemployed, percentage without health insurance, and population density (population per 100 square miles), were obtained from the US Census Bureau’s 2021 American Community Survey (5-year estimates).^19^

### Study Population

This study included female homicide decedents of child-bearing age (15-49 years). Although the term *women* is not inclusive of all individuals who become pregnant, we use this term as used in the NVDRS. The study population was stratified into 2 subgroups: nonpregnant and pregnant or pregnant in the last 12 months (henceforth termed *pregnant*), as categorized in the NVDRS. Women with unknown pregnancy status were included in the nonpregnant group. For quality assurance, we excluded 13 states in which female homicide rates in the NVDRS were less than 85% of what was reported in WISQARS (indicating incomplete case capture in those states by NVDRS data), leaving 37 states for analysis (eTable 1 in Supplement 1). We used the race and ethnicity categories as coded in the NVDRS, which is based on information from law enforcement, medical examiner and coroner reports, and/or death certificates. The “other” category combined the NVDRS classifications “other/unspecified” and “more than one race.”^20^ The data were used descriptively to examine population-level disparities in pregnancy-associated homicide.

### Measures and Outcomes

The primary outcomes were state-level all-cause and firearm-specific homicide rates, stratified by pregnancy status. Homicide rates were calculated per 1 000 000 live births for pregnant women and per 1 000 000 adult women aged 15 to 49 years (excluding live births) for nonpregnant women. The primary exposure was state-level firearm ownership prevalence. This was estimated using the ratio of firearm suicides to total suicides, a proxy used in prior research because specific data on state-level firearm ownership prevalence were not available for the years under study.^15,16^ Additional state-level covariates (age, sex, poverty, unemployment, insurance, and population density) were obtained for inclusion in the multivariable regression modeling to account for socioeconomic differences across states. These covariates have been previously reported to be associated with firearm access, homicide risk, and/or maternal outcomes and/or have been used in prior firearm research literature.^21,22,23,24,25^

### Statistical Analysis

We calculated frequencies of demographic, incident, and suspect characteristics, stratified by pregnancy status. We then calculated state-level pregnancy-associated all-cause and firearm-specific homicide rates using live births as the denominator, aggregated for the full study period. For descriptive comparison, we also calculated national all-cause and firearm-specific homicide rates among nonpregnant women, using WISQARS population estimates of women aged 15 to 49 years (excluding live births) as the denominator.

To test the association between estimated state-level firearm ownership and pregnancy-associated homicide, we estimated a series of multivariable generalized estimating equations with the negative binomial family and log link, with the state-year as the unit of analysis, reporting adjusted incidence rate ratios (IRRs) and 95% CIs. The dependent variable was the state-year homicide count, with the number of live births as the offset. Separate models were estimated for all-cause and firearm-specific pregnancy-associated homicides. The state-year firearm ownership proxy was the independent variable, with a 1-unit change corresponding to a 1% change in state-level ownership percentage. In addition to calendar year, the following state-level population factors were included as covariates in the model: percentage aged 15-29 years, percentage male, percentage below the poverty level, percentage unemployed, percentage without health insurance, and population density (mean population per 100 square miles). Because the unit of analysis was the state-year, not the individual, individual-level demographic variables (eg, age, race, and ethnicity) were not included in the regression models. All models used robust SEs and an unstructured within-panel (ie, state) correlation structure. We also conducted a sensitivity analysis excluding the 4 states with no recorded pregnancy-associated firearm homicides (Oklahoma, Rhode Island, New Hampshire, and Vermont) to assess the robustness of potential underreporting. Data were collected and analyzed between November 2023 and July 2025. Statistical significance was defined as a 2-sided *P *< .05. All analyses were conducted using Stata, version 19.0 (StataCorp).

## Results

Between 2018 and 2021, 7063 homicides occurred among women aged 15 to 49 years (median [IQR] age, 30.0 [24.0-38.0] years; 153 [2.2%] American Indian or Alaska Native, 115 [1.6%] Asian or Pacific Islander, 3278 [46.4%] Black or African American, 2565 [36.3%] White, 123 [1.7%] other [other or unspecified race and >1 race], and 29 [0.4%] unknown) across 37 states included in this analysis. Of these women who died by homicide, 434 (6.1%) were pregnant and 6629 (93.9%) were nonpregnant. The highest proportion of homicides occurred among pregnant women aged 20 to 24 years (141 [32.5%]), whereas for nonpregnant women, the highest proportion was among those aged 25 to 29 years (1229 [18.5%]). By race and ethnicity, non-Hispanic Black women accounted for the highest proportion of homicides for both pregnant (250 [57.6%]) and nonpregnant (3028 [45.7%]) women. Most incidents involved firearms for both pregnant (341 [78.6%]) and nonpregnant (4787 [72.2%]) women, particularly handguns. The most common locations of death among pregnant women were the home (149 [34.3%]) and emergency department and outpatient settings (102 [23.5%]) compared with nonpregnant women for whom the most common locations were other locations (2364 [35.7%]) and home (2101 [31.7%]). Suspected perpetrators were most often male: 303 (86.8%) for pregnant and 4247 (81.1%) for nonpregnant women (Table 1).

**Table 1.** Demographic, Incident, and Suspect Characteristics of Homicide Among Women of Childbearing Age, United States, 2018-2021

Characteristic	No. (%) of women		
	Pregnant (n = 434)^a^	Nonpregnant (n = 6629)^b^	Total (N = 7063)
Demographic characteristics			
Age group, y			
15-19	59 (13.6)	696 (10.5)	755 (10.7)
20-24	141 (32.5)	1087 (16.4)	1228 (17.4)
25-29	98 (22.6)	1229 (18.5)	1327 (18.8)
30-34	94 (21.7)	1068 (16.1)	1162 (16.5)
35-39	36 (8.3)	988 (14.9)	1024 (14.5)
40-44	6 (1.4)	850 (12.8)	856 (12.1)
45-49	0	711 (10.7)	711 (10.1)
Age, median (IQR), y	26.0 (21.0-30.0)	31.0 (24.0-39.0)	30.0 (24.0-38.0)
Race and ethnicity			
Hispanic	46 (10.6)	754 (11.4)	800 (11.3)
Non-Hispanic American Indian or Alaska Native	7 (1.6)	146 (2.2)	153 (2.2)
Non-Hispanic Asian or Pacific Islander	9 (2.1)	106 (1.6)	115 (1.6)
Non-Hispanic Black	250 (57.6)	3028 (45.7)	3278 (46.4)
Non-Hispanic White	106 (24.4)	2459 (37.1)	2565 (36.3)
Missing	1 (0.2)	28 (0.4)	29 (0.4)
Other^c^	15 (3.5)	108 (1.6)	123 (1.7)
US Census region			
Northeast	42 (9.7)	673 (10.2)	715 (10.1)
Midwest	132 (30.4)	2327 (35.1)	2459 (34.8)
South	202 (46.5)	2686 (40.5)	2888 (40.9)
West	58 (13.4)	943 (14.2)	1001 (14.2)
Educational level			
Less than high school	106 (24.4)	1582 (23.9)	1688 (23.9)
High school or GED	208 (47.9)	2805 (42.3)	3013 (42.7)
Some college	87 (20.0)	1586 (23.9)	1673 (23.7)
Bachelor’s degree	16 (3.7)	424 (6.4)	440 (6.2)
Graduate degree	7 (1.6)	141 (2.1)	148 (2.1)
Missing	10 (2.3)	91 (1.4)	101 (1.4)
Incident characteristics			
Place of death			
Inpatient	39 (9.0)	744 (11.2)	783 (11.1)
ED or outpatient setting	102 (23.5)	1255 (18.9)	1357 (19.2)
Hospice	0	3 (0.0)	3
Nursing home or long-term care facility	1 (0.2)	6 (0.1)	7 (0.1)
Dead on arrival	7 (1.6)	91 (1.4)	98 (1.4)
Home	149 (34.3)	2101 (31.7)	2250 (31.9)
Other	131 (30.2)	2364 (35.7)	2495 (35.3)
Unknown	5 (1.2)	65 (1.0)	65 (0.9)
Weapon used			
Firearm	341 (78.6)	4787 (72.2)	5128 (72.6)
Sharp instrument	42 (9.7)	811 (12.2)	853 (12.1)
Blunt instrument	12 (2.8)	242 (3.7)	254 (3.6)
Hanging or strangulation	22 (5.1)	296 (4.5)	318 (4.5)
Other	12 (2.8)	341 (5.1)	353 (5.0)
Missing	5 (1.2)	152 (2.3)	157 (2.2)
Firearm type used			
Handgun	207 (60.7)	2728 (57.0)	2935 (57.2)
Long gun	29 (8.5)	370 (7.7)	399 (7.8)
Other	0	3 (0.1)	3 (0.1)
Unknown	105 (30.8)	1688 (35.2)	1793 (35.0)
Suspect characteristics			
Age, median (IQR), y	33.0 (25.0-42.0)	27.0 (22.0-33.0)	32.0 (25.0-41.0)
Sex			
Male	303 (86.8)	4247 (81.1)	4550 (81.5)
Female	14 (4.0)	340 (6.5)	354 (6.3)
Unknown	32 (9.2)	650 (12.4)	682 (12.2)
Race^d^			
American Indian or Alaska Native	1 (0.3)	42 (0.8)	43 (0.7)
Asian or Pacific Islander	5 (1.4)	51 (0.9)	56 (1.0)
Black or African American	193 (53.9)	2112 (39.0)	2305 (39.9)
White	71 (19.8)	1520 (28.1)	1591 (27.5)
Other^c^	70 (16.1)	1239 (18.7)	1309 (18.5)
Unknown	18 (5.0)	453 (8.4)	471 (8.2)

Abbreviations: ED, emergency department; GED, General Educational Development.

^a^ Pregnant homicides are defined as occurring among women who were pregnant or pregnant within the past 1 year before death, as categorized by the National Violent Death Reporting System.

^b^ Nonpregnant homicides are defined as occurring among women who were not pregnant or whose pregnancy status was unknown by the National Violent Death Reporting System.

^c^ Other or unspecified or more than 1 race.

^d^ The National Violent Death Reporting System dataset did not include suspect ethnicity.

**Figure 1.**
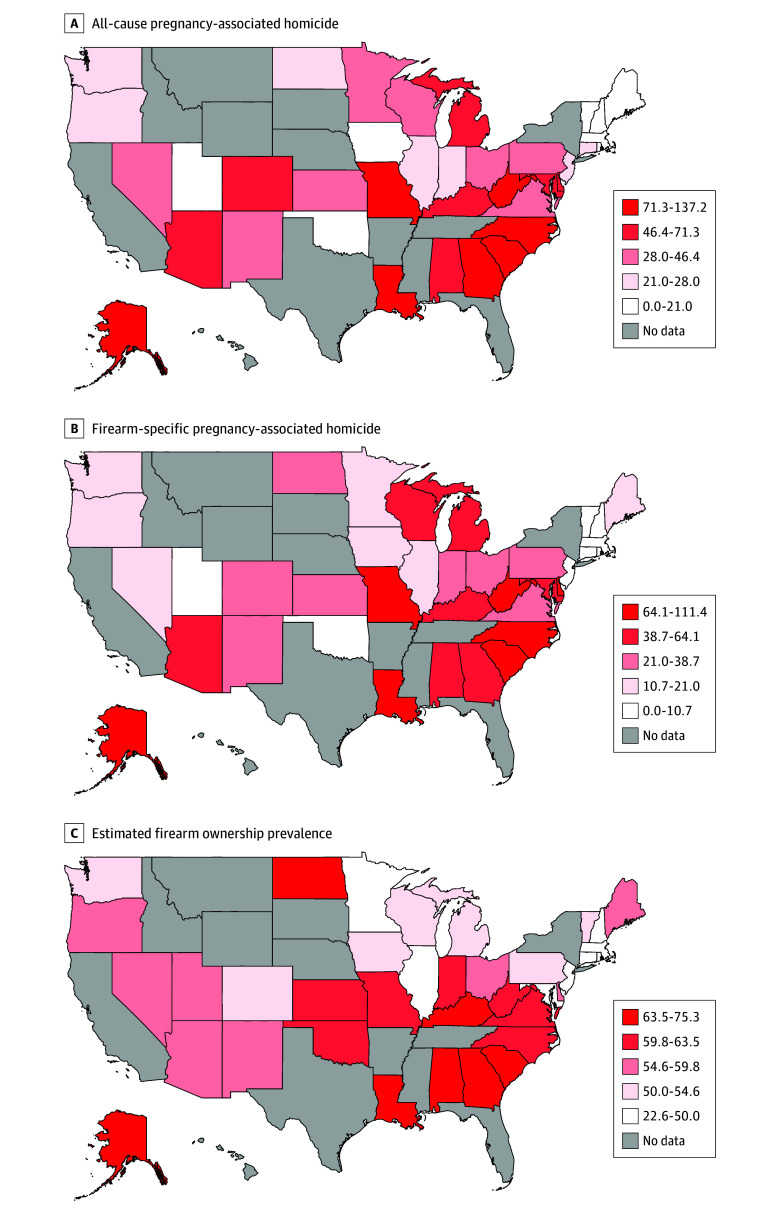
State-Level Pregnancy-Associated Homicide Rates, Firearm-Specific Homicide Rates, and Estimated Firearm Ownership Rates in the US, 2018-2021 Homicide rates are per 1 000 000 live births. Firearm ownership, reported as firearm ownership per 100 households, was estimated using the proxy measure of the ratio of firearm suicides to total suicides. States excluded from analysis due to data quality thresholds are shown in gray.

Nationally, the all-cause pregnancy-associated homicide rate (49.16 per 1 000 000 live births) exceeded that among nonpregnant women (39.05 per 1 000 000 women) (IRR, 1.26; 95% CI, 1.14-1.39). Firearm-specific homicide rates were also greater in pregnant (38.63 per 1 000 000 live births) compared with nonpregnant individuals (28.20 per 1 000 000 women) (IRR, 1.37; 95% CI, 1.22-1.53). State-level all-cause homicide rates for pregnant women ranged from 0 per 1 000 000 live births (New Hampshire, Rhode Island and Vermont) to 137.15 per 1 000 000 live births (Louisiana) (Table 2, Figure 1A). Firearm-specific state-level homicide rates for pregnant women ranged from 0 per 1 000 000 live births (New Hampshire, Oklahoma, Rhode Island, Vermont) to 111.43 per 1 000 000 live births (Louisiana) (Table 2, Figure 1B). Firearms accounted for more than 50% of pregnancy-associated homicides in 29 of 33 states (87.9%) with nonzero firearm homicide data: in 21 of 33 states (63.6%), 75% or more of pregnancy-associated homicides involved firearms, and in 11 states (33.3%), 90% or more involved firearms (Table 2). Estimated state-level firearm ownership prevalence varied widely across the states (Figure 1C).

**Table 2.** State-Level Homicide Rates of Pregnant Women Stratified by Use of Firearm

State	All-cause pregnancy homicides per 1 000 000 live births	Firearm pregnancy homicides per 1 000 000 live births	Estimated firearm ownership (FS/S ratio)
Louisiana	137.15	111.43	0.65
Missouri	123.18	109.10	0.61
Alaska	103.24	77.43	0.61
West Virginia	98.72	70.52	0.62
South Carolina	88.26	70.61	0.64
Georgia	88.16	64.12	0.63
North Carolina	82.13	71.60	0.60
Delaware	71.33	71.33	0.49
Michigan	60.89	56.21	0.52
Alabama	60.32	47.40	0.70
Colorado	55.96	27.98	0.51
Arizona	53.97	41.27	0.58
Kentucky	52.16	52.16	0.63
National	49.16	38.63	0.44
Maryland	46.75	43.15	0.58
Virginia	46.41	38.68	0.52
Pennsylvania	45.01	31.88	0.52
New Mexico	44.80	22.40	0.56
Wisconsin	44.05	40.04	0.51
Ohio	37.84	35.95	0.54
Nevada	36.21	14.48	0.58
Kansas	35.53	35.53	0.57
Minnesota	30.62	19.14	0.46
Indiana	28.03	21.80	0.59
Illinois	25.43	19.98	0.40
New Jersey	24.98	9.99	0.26
Oregon	24.27	12.14	0.53
North Dakota	24.24	24.24	0.58
Washington	23.67	11.84	0.50
Connecticut	21.72	7.24	0.29
Maine	20.99	20.99	0.55
Iowa	20.22	20.22	0.48
Massachusetts	14.61	7.30	0.22
Utah	10.73	10.73	0.52
Oklahoma	5.13	0	0.60
New Hampshire	0	0	0.50
Rhode Island	0	0	0.28
Vermont	0	0	0.55

**Figure 2.**
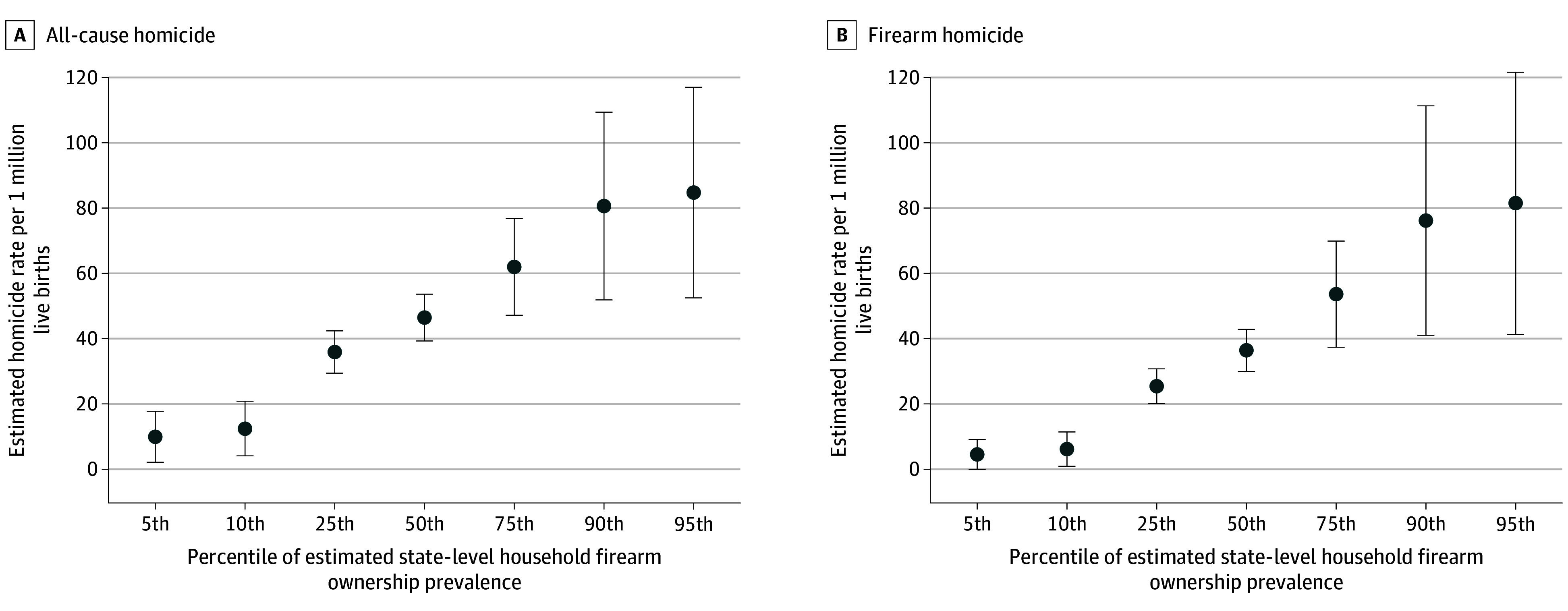
Adjusted Associations Between State-Level Estimated Firearm Ownership and Pregnancy-Associated Homicide Rates Estimated rates of all-cause and firearm-specific pregnancy-associated homicide per 1 000 000 live births across percentiles of state-level firearm ownership, estimated using the ratio of firearm suicides to total suicides. Estimates are derived from the multivariable negative binomial regression model adjusted for year and state percentage of the population for age, sex, poverty, unemployment, insurance coverage, and population density. Error bars represent 95% CIs.

In the adjusted multivariable models, every 1% increase in the estimated state-level firearm ownership prevalence was significantly associated with a 6% increase in the pregnancy-associated all-cause homicide rate (adjusted IRR, 1.06; 95% CI, 1.03-1.09) and an 8% increase in firearm homicide (adjusted IRR, 1.08; 95% CI, 1.04-1.12). In these adjusted models, there was a stepwise increase in estimated rates of both all-cause and firearm homicide of pregnant women across the range of estimated state-level firearm ownership prevalence (Figure 2). No other state-level variables were significantly associated with pregnancy-associated homicide (Table 3).

**Table 3.** Multivariable Analysis of State-Level Variables of All-Cause and Firearm Homicide Among Pregnant Women^a^

State-level independent variable	Adjusted IRR (95% CI)	
	All-cause mortality	Firearm mortality
Owning firearms^b^	1.06 (1.03-1.09)	1.08 (1.04-1.12)
Year		
2018	1 [Reference]	1 [Reference]
2019	1.40 (0.95-2.06)	1.43 (0.91-2.24)
2020	1.30 (0.89-1.89)	1.37 (0.88-2.14)
2021	1.29 (0.88-1.91)	1.25 (0.80-1.95)
Percentage aged 15-29 y	0.94 (0.79-1.12)	1.03 (0.85-1.24)
Percentage male	0.95 (0.74-1.22)	0.80 (0.55-1.16)
Percentage living below federal poverty level	0.98 (0.89-1.09)	0.97 (0.86-1.09)
Percentage unemployed	1.23 (0.93-1.65)	1.16 (0.82-1.66)
Percentage uninsured	0.99 (0.89-1.09)	0.93 (0.84-1.04)
Population density	1.06 (0.98-1.14)	1.05 (0.95-1.15)

Abbreviation: IRR, incidence rate ratio.

^a^ A total of 13 states were excluded due to data quality concerns: Arkansas, California, Florida, Hawaii, Idaho, Mississippi, Montana, Nebraska, New York, South Dakota, Tennessee, Texas, Wyoming.

^b^ Estimated state-level firearm prevalence as measured by the ratio of firearm suicides to total suicides.

Sensitivity analyses excluding 4 states with no recorded pregnancy-associated homicides (New Hampshire, Oklahoma, Rhode Island, and Vermont) yielded similar results. In these models, state-level estimated firearm ownership prevalence remained significantly associated with all-cause homicide (adjusted IRR, 1.05; 95% CI, 1.02-1.07) and firearm homicide (adjusted IRR, 1.06; 95% CI, 1.03-1.10) (eTable 2 in Supplement 1). Associations for other covariates remained nonsignificant.

## Discussion

In this retrospective cross-sectional study of more than 7000 homicides among women of child-bearing age from 2018 to 2021, pregnant compared with nonpregnant women were at increased risk for all-cause homicide, largely driven by firearm homicide. Among pregnant women, every 1% increase in state-level firearm ownership was significantly associated with a 6% increase in all-cause homicide and an 8% increase in firearm homicide, even after adjusting for other state-level factors. Our study, to our knowledge, is novel in evaluating how broader structural factors, including state-level firearm prevalence and other state-level factors, are associated with the risk of firearm homicide among women during pregnancy. These results suggest that even incremental increases in firearm availability may contribute to measurable increases in homicide risk in pregnant women.

Firearm homicide is the leading cause of death for pregnant women—beyond medical causes, including eclampsia and hemorrhage.^3^ Prior work^11^ has shown that pregnancy is a time of elevated homicide risk, often in the setting of intimate partner violence. In our study population, pregnant women experienced a 37% higher firearm homicide rate compared with nonpregnant women. Firearms were the leading mechanism of pregnancy-associated homicide, accounting for 78.6% of the weapons used for homicide. This pattern was consistent across states, with 75% or more of pregnancy-associated homicides involving firearms in two-thirds of the states. These findings highlight the critical role of firearm access in pregnancy-associated homicide.

Among the pregnant women who died by homicide, there were stark demographic differences. By age group, the highest proportion of homicides in pregnant women was among those aged 20 to 24 years, whereas the highest proportion of homicides in nonpregnant women was among those aged 25 to 29 years. There were disparities by race and ethnicity, with the highest proportion of homicides for both pregnant and nonpregnant women among individuals of non-Hispanic Black race. These trends are consistent with prior work showing that pregnancy-associated homicide disproportionately affects young, non-Hispanic Black women.^10,26^ These demographic patterns reflect longstanding inequities and highlight the compounded risk of homicide at the intersection of youth, pregnancy, socioeconomic disadvantage, and race. Existing firearm violence research similarly emphasizes the role of structural disadvantage in shaping risk factors for experiencing violence.^27,28^ Our findings suggest that risks of pregnancy-associated homicide are shaped not only by individual factors but also by broader systems of inequity.

We also observed large geographic differences in pregnancy-associated homicide rates among states. Although several states had zero homicides in pregnant women during the study period, other states had more than 100 deaths of pregnant women per 1 million live births. This variation raises important questions about why the risk of homicide during pregnancy differs so substantially based on place of residence. In our multivariable models adjusting for multiple state-level factors, only estimated state-level firearm ownership prevalence, using an established proxy measure,^15,16^ demonstrated a statistically significant association with the outcome of firearm homicide IRR among pregnant women. There was a stepwise increase in the firearm homicide incidence rate among pregnant women across the range of estimated state-level firearm ownership prevalence. This association persisted even after adjusting for other state-level characteristics, which reinforces firearm availability as a key environmental risk factor for homicide in pregnant women.

There are important policy implications from the findings of our study. The observed geographic disparities likely reflect differences in state-level firearm-related legislation as well as policies regarding access to comprehensive reproductive health care. In the general population, increased firearm ownership prevalence is associated with increased firearm fatalities.^13,14^ States with more restrictive firearm laws are associated with decreased firearm deaths.^29,30,31^ Policies that reduce community-level access to firearms, such as safe storage laws or domestic violence firearm prohibitions, may play an essential role in the protection of pregnant women. Specifically, firearm legislation, which includes domestic violence–related relinquishment laws, is associated with a reduction in pregnancy-associated homicides.^32,33^ Policies related to comprehensive reproductive care are also important to consider in addressing homicide and firearm homicide in pregnant women. Prior research has found higher rates of intimate partner–related female homicide, and specifically peripartum homicide, in states with more restrictive abortion laws.^34,35^ One possible explanation for this association is that limitations on access to abortions potentially result in continuation of an unplanned pregnancy, thus increasing the potential for fatal violence from intimate partners or family members when the pregnancy is unplanned. As with firearm policy, these findings suggest that reproductive health care policy may represent a critical point of intervention to reduce pregnancy-associated homicide. Future research is needed to better understand how specific state-level firearm and reproductive health care access policies, structural inequities, and the social determinants of health intersect to shape homicide risk during pregnancy. Improved surveillance of pregnancy-associated homicide, including more complete reporting of pregnancy status, will also be critical for research efforts. This knowledge could inform policy efforts to address the increased homicide risk among pregnant women. Given the unique risks for this population, health care professionals involved in prenatal, emergency, and postpartum care should also integrate firearm safety as part of intimate partner violence screening during medical care.

### Limitations

This study has several limitations. There is the risk of pregnancy status misclassification and/or misclassification of manner of death (eg, homicide vs unintentional) when using a large national database due to variability in methods of determination as well as potential miscoding. There were missing data, including the pregnancy status. Individuals for whom pregnancy status was unknown were included in the nonpregnant group for analysis purposes, which would bias results toward the null by misclassifying some pregnant women as nonpregnant. Gender identity is also not routinely captured in NVDRS, limiting inclusion to individuals identified as women. Next, we used the state firearm suicide to total suicide ratio as a proxy for state-level firearm ownership prevalence, which may introduce bias. Recent work suggests the proxy may overestimate household firearm ownership among minority groups and unmarried individuals and underestimate it in others due to variation in the use of firearms for suicide across regions and populations.^36^ However, in the absence of primary data collection for state firearm ownership, this method is highly correlated with firearm ownership and has been used by multiple prior studies.^15,16^ Scaling homicide rates to per 1 000 000 individuals improved interpretability but may overstate the perceived risk, especially in smaller states where rates may reflect only 2 or 3 deaths annually. Nationally, the absolute risk of pregnancy-associated firearm homicide remains low (approximately 36 deaths per 1 000 000 live births), suggesting the need for caution when interpreting state-level differences. The study is also limited by its use of state-level analyses, which preclude the inclusion of individual-level characteristics, such as individual age, race, and ethnicity, in our models. In addition, although we adjusted for multiple known state-level covariates, which are associated with firearm mortality risk based on previous literature,^21,22,23,24,25^ unmeasured confounders, such as intimate partner violence prevalence and racial inequities, may remain. We also excluded from analysis states where the NVDRS female homicide counts were less than 85% of CDC-reported values due to concerns about data quality; however, this may limit generalizability of our findings. Lastly, our 4-year study period limits assessment of longer-term trends. Future studies using longer periods should be conducted to better understand the associations explored here.

## Conclusions

Homicide remains the leading cause of maternal mortality in the US. With more than three-quarters of these deaths caused by firearms, firearm homicide must be considered a maternal health crisis. Our findings demonstrate that pregnant women are at increased risk for firearm homicide compared with their nonpregnant peers. Increased state-level firearm ownership prevalence is statistically associated with increased state-level firearm homicide incidence rates among pregnant women. These deaths are not random. They are predictable and therefore preventable. Preventing homicide during pregnancy will require urgent and coordinated actions from policymakers, public health advocates, and health care systems to address this leading cause of death in pregnant women.

## Notes

Supplement 1. **eTable 1. **State-Level Data Quality Metric **eTable 2. **Predictors of All-Cause and Firearm Homicide Against Pregnant Victims, Excluding States With No Recorded Pregnancy Homicides

Supplement 2.
Data Sharing Statement
