# Supplementary material for: Firearm Homicide in Pregnant Women and State-Level Firearm Ownership
Source: JAMA Netw Open. 2025 Nov 10;8(11):e2542447. doi: 10.1001/jamanetworkopen.2025.42447 (PMC12603857; doi:10.1001/jamanetworkopen.2025.42447)
Supplement: Supplement 1. — eTable 1. State-Level Data Quality Metric eTable 2. Predictors of All-Cause and Firearm Homicide Against Pregnant Victims, Excluding States With No Recorded Pregnancy Homicides [file jamanetwopen-e2542447-s001.pdf]

Supplemental Online Content

Dholakia A, Monuteaux MC, D'Ambrosi G, McLone SG, Fleegler E, Lee LK. Firearm homicide in pregnant women and state-level firearm ownership. *JAMA Netw Open*. 2025;8(11):e2542447. doi:10.1001/jamanetworkopen.2025.42447

eTable 1. State-Level Data Quality Metric

eTable 2. Predictors of All-Cause and Firearm Homicide Among Pregnant Victims, Excluding States With No Recorded Pregnancy Homicides

This supplemental material has been provided by the authors to give readers additional information about their work.

© 2025 Dholakia A et al. *JAMA Network Open*.

eTable 1. State-Level Data Quality Metric[a]

Included States	Percent Coverage	Excluded States	Percent Coverage
Alabama	102.69	Arkansas	49.73
Alaska	96.08	California	58.89
Arizona	93.73	Florida[c]	n/a
Colorado	96.77	Hawaii	27.27
Connecticut	104.92	Idaho	47.62
Delaware	97.83	Mississippi	42.65
Georgia	97.91	Montana	55.26
Illinois	90.54	Nebraska	84.21
Indiana	98.51	New York	78.10
Iowa	114.06	South Dakota	69.57
Kansas	95.15	Tennessee	61.33
Kentucky	104.81	Texas	34.29
Louisiana	99.42	Wyoming	80.00
Maine	113.64		
Maryland	101.96		
Massachusetts	95.24		
Michigan	100.78		
Minnesota	94.06		
Missouri	99.75		
Nevada	103.57		
New Hampshire	85.00		
New Jersey	95.15		
New Mexico	100.71		
North Carolina	104.39		
North Dakota	100.00		
Ohio	98.37		
Oklahoma	107.43		
Oregon	97.30		
Pennsylvania	85.89		
Rhode Island	87.50		
South Carolina	100.69		
Utah	96.36		
Vermont[b]	n/a[1]		
Virginia	98.83		
Washington	97.19		
West Virginia	111.11		
Wisconsin	96.88		

[a]Percent of state-level deaths documented in WISQARS (https://wisqars.cdc.gov/) that were represented in NVDRS data (https://www.cdc.gov/nvdrs/about/index.html)
[b]Vermont had zero recorded deaths during the study period, so percent coverage could not be calculated
[c]Florida did not report data in NVDRS for the study period due to incompleteness, so percent coverage could not be calculated

© 2025 Dholakia A et al. *JAMA Network Open*.

eTable 2. Predictors of all-cause and firearm homicide among pregnant victims, excluding states with no recorded pregnancy homicides[a]

State-Level Independent Variables	All-Cause Homicide aIRR (95% CI)	Firearm Homicide aIRR (95% CI)
**Percent owning firearms[b]**	**1.05 (1.02, 1.07)**	**1.06 (1.03, 1.10)**
**Year**		
**2018**	referent	referent
**2019**	1.39 (0.94, 2.06)	1.44 (0.91, 2.27)
**2020**	1.37 (0.95, 1.97)	1.44 (0.93, 2.23)
**2021**	1.38 (0.95, 1.99)	1.37 (0.89, 2.10)
**Percent aged 15-29 years**	0.91 (0.76, 1.09)	0.99 (0.81, 1.20)
**Percent male**	1.01 (0.81, 1.26)	0.86 (0.61, 1.23)
**Percent living below federal poverty level**	1.02 (0.95, 1.10)	1.02 (0.91, 1.14)
**Percent unemployed**	1.09 (0.86, 1.39)	1.02 (0.74, 1.42)
**Percent uninsured**	1.03 (0.96, 1.11)	0.99 (0.90, 1.08)
**Population density**	1.06 (0.99, 1.15)	1.05 (0.95, 1.16)

[a]13 states excluded due to data quality concerns: Arkansas, California, Florida, Hawaii, Idaho, Mississippi, Montana, Nebraska, New York, South Dakota, Tennessee, Texas, Wyoming
4 states excluded due to no recorded pregnancy homicides: New Hampshire, Oklahoma, Rhode Island, Vermont
[b]Estimated state-level firearm prevalence as measured by the ratio of firearm suicides to total suicides (FS/S)

© 2025 Dholakia A et al. *JAMA Network Open*.
