# Supplementary material for: Firearm Homicide in Pregnant Women and State-Level Firearm Ownership
Source: JAMA Netw Open. 2025 Nov 10;8(11):e2542447. doi: 10.1001/jamanetworkopen.2025.42447 (PMC12603857; doi:10.1001/jamanetworkopen.2025.42447)
Supplement: Supplement 2. — Data Sharing Statement [file jamanetwopen-e2542447-s002.pdf]

Data Sharing Statement

Dholakia. Firearm Homicide in Pregnant Women and State-Level Firearm Ownership. *JAMA Netw Open*. Published November 10, 2025. doi:10.1001/jamanetworkopen.2025.42447

Data
Data available: Yes
Data types: Deidentified participant data
How to access data: The data used in this study were obtained from the National Violent Death Reporting System (NVDRS) Restricted Access Database (RAD). Researchers can apply for access to this data through the CDC's NVDRS Restricted Access Data Request process.
When available: With publication

Supporting Documents
Document types: Statistical/analytic code
How to access documents: The statistical code and analytical methods used in this study are available upon request to the corresponding author (Ayesha Dholakia, Ayesha.Dholakia@childrens.harvard.edu).
When available: With publication

Additional Information
Who can access the data: This data will be made available to researchers whose proposed use of the data has been approved.
Types of analyses: This data will be made available for a specified purpose e.g., pursuance of a project utilizing similar research methods.
Mechanisms of data availability: The data will be made available after discussion and approval by this research team.
